# Saliva in the diagnosis of diseases

**DOI:** 10.1038/ijos.2016.38

**Published:** 2016-09-02

**Authors:** Chen-Zi Zhang, Xing-Qun Cheng, Ji-Yao Li, Ping Zhang, Ping Yi, Xin Xu, Xue-Dong Zhou

**Affiliations:** 1 State Key Laboratory of Oral Diseases, West China Hospital of Stomatology, Sichuan University, Chengdu, China; 2 Department of Operative Dentistry and Endodontics, West China Hospital of Stomatology, Sichuan University, Chengdu, China; 3 The Clinical Laboratory of West China Hospital of Stomatology, Sichuan University, Chengdu, China

**Keywords:** diagnosis, oral diseases, precision medicine, saliva, systemic diseases

## Abstract

Saliva is secreted from the salivary glands and has multiple functions, including mouth cleaning and protection, antibacterial effects and digestion. With the rapid advancement in salivaomics, saliva is well recognized as a pool of biological markers. Saliva, as a non-invasive and safe source, could be a substitute for blood in the diagnosis and prognosis of diseases. This review summarizes the latest advancements in saliva-related studies and addresses the potential value of saliva in the early diagnosis of oral diseases, such as dental caries and periodontal disease, as well as cancer, diabetes and other systemic disorders. Saliva biomarkers range from changes in the biochemical indices of DNA, RNA and proteins to the diversification of microbiota structures. This study integrates data reported in the recent literature and discusses the clinical significance and prospects for the application of saliva in the early diagnosis of diseases, translational medicine and precision medicine.

## Introduction

Saliva is a hypotonic solution of salivary acini, gingival crevicular fluid and oral mucosal exudates. Approximately 90% of saliva is secreted from the salivary glands and the major glands include the parotid glands, submandibular glands and sublingual glands. The salivary glands with high permeability are surrounded by abundant capillaries, blood and acini, and can exchange molecules. Thus, biomarkers in the blood circulation can infiltrate acini and eventually be secreted into the saliva. Saliva is colourless, odourless and has a relative density of 1.004–1.009 and a pH of 6.6–7.1. A normal person produces 600 mL of saliva per day. Saliva consists of 99% water and the remainder is organic molecules such as salivary amylase, mucopolysaccharide, mucin and lysozymes, and some inorganic matter such as Na^+^, K^+^, Ca^2+^, Cl^−^ and the thiocyanate ion. Saliva has multiple functions as follows: first, it helps to clean the mouth by washing away bacteria or food residues and freshening the breath; second, salivary amylase, a form of amylase in the saliva of human beings, catalyses the hydrolysis of starch into maltose and sometimes glucose in the mouth; third, lysozymes and thiocyanate ions in the saliva are bactericidal, making saliva an important part of the nonspecific immune system of humans; and fourth, saliva is secretory and contains risk factors for some diseases by excreting or transmitting KI, Pb and Hg, and viruses such as rabies, polio and human immune deficiency virus (HIV). In the past, doctors have diagnosed diseases with the use of serum or urine tests, which are either painful or embarrassing, respectively. However, saliva is now considered a potential pool of biological markers that range from changes in biochemicals, DNA, RNA and proteins to the microbiota structure. It is relatively safe to collect saliva and minimizes the risk of virus spread. Hence, saliva provides a new, non-invasive and simple way to help in the diagnosis of disease, and it is expected to become a substitute for serum or urine tests in disease diagnosis.

## Saliva constituents

Saliva has a complex composition that includes urea, ammonia, uric acid, glucose, cholesterol, fatty acid, triglycerides, neutral lipid, glycolipid, amino acid, steroid hormones, mucin, amylase, lectin, glycoprotein, lysozyme, peroxidase and lactoferrin. It also contains high concentrations of Na^+^, Cl^–^, Ca^2+^, K^+^, HCO_3_^–^, H_2_PO_4_^–^, F^–^, I^–^ and Mg^2+^ from the serum. In addition, saliva contains >700 microorganisms that are related to oral and systemic diseases.

Owing to the rapid progress made in salivary studies, researchers have proposed the concept of salivaomics. Salivaomics encompasses genomics, transcriptomics, proteomics, metabonomics and microRNA (miRNA) analysis. Wong^1^ built a professional Salivaomics Knowledge Base (SKB) that can systematically manage the data of research related to salivaomics. The SKB (http://www.hspp.ucla.edu/skb.swf) is currently the only website that is devoted to research on salivaomics and it has collected a large amount of information related to salivaomics, pharmacoproteomics, pharmacogenomics and similar fields. It has been well recognized that salivary biomarkers can be exploited for the early diagnosis of some oral and systemic diseases.

## Diagnosis of oral diseases by saliva

### Caries

The prevalence of dental caries is positively correlated with the microbial load of *Streptococcus mutans* and *Lactobacillus* in the saliva. Samaranayake^2^ used paraffin wax to stimulate the production of the salivary samples, which were then incubated in a selective growth media for up to 24 h. Saliva from populations with high caries activity contained >1 × 10^6^ mL^−1^ of *S. mutans* and/or 1 × 10^5^ mL^−1^ of *Lactobacillus*. Saliva from populations with low caries activity harboured <1 × 10^5^ mL^−1^ of *S. mutans* and/or 1 × 10^4^ mL^−1^ of *Lactobacillus*. The structures and functions of salivary bacteria have been studied as potential predictive markers for caries onset. Yang *et al.*^3^ analysed adult saliva microbiomes in 19 caries-active and 26 healthy human hosts by whole-genome-based deep sequencing and cross-validated 16S rRNA amplicon-based technologies. They observed an overabundance of the *Prevotella* genus in the caries microbiota compared with healthy ones. In addition, *Prevotella* species differed in caries-active and normal individuals, indicating the predictive role of *Prevotella* in the onset of dental caries. Another study from the same group established the first model, known as Microbial Indicators of Caries, to diagnose caries and predict potential caries onset for samples clinically considered healthy. The accuracy of the prediction using the *Prevotella* genus and microbiota is similar, validating the idea that the *Prevotella* genus is of great significance for the timely prediction of caries.^4^

### Periodontal diseases

*Porphyromonas gingivalis* is a ‘red complex' bacteria that is closely associated with periodontitis. Recently, some researchers developed an enzyme-linked immunosorbent assay-based *P. gingivalis* saliva kit to specifically detect this bacterium in saliva. The kit can detect both laboratory and clinical isolate strains of *P. gingivalis* at concentrations of 5 × 10^4^ to 5 × 10^5^ CFU·mL^−1^ and yields results within 90 s. Compared with real-time polymerase chain reaction technology, the *P. gingivalis* saliva kit is rapid and has a sensitivity of 92% and a specificity of 96%. Therefore, the *P. gingivalis* saliva kit is expected to be an easy and time-efficient chair-side diagnostic tool for the detection of *P. gingivalis*.^5^ A recent study detected the levels of adrenomedullin (AM) and nitric oxide (NO) in saliva and gingival crevicular fluid collected from patients with gingivitis, aggressive periodontitis and chronic periodontitis, and compared them with the healthy controls. Salivary AM and NO levels distinguished patients with aggressive periodontitis from other groups. In contrast, patients with chronic periodontitis, aggressive periodontitis and gingivitis showed increased levels of NO in the gingival crevicular fluid and the levels of AM were higher in patients with periodontitis compared with those with only gingivitis. These data indicate a functional linkage between NO and AM in periodontal disease, and AM and NO could be used as salivary diagnostic markers for periodontitis.^6^ Data from another study demonstrated that NO and its end metabolites in saliva are more valuable for the diagnosis of periodontal diseases than gingival crevicular fluid.^7^ Tobacco use is regarded as a major risk factor for periodontal diseases. A recent study showed positive correlations between salivary superoxide dismutase levels and clinical symptoms such as gingival index, pocket depth and clinical attachment loss in patients with chronic periodontitis. The study also demonstrated the potential of saliva as a more convenient and non-invasive way to diagnose patients with higher risks for precancerous lesions and conditions.^8^ Salivary macrophage inflammatory protein-1α, matrix metalloproteinase-8, interleukin (IL)-1β, IL-6, prostaglandin E2 and tumour necrosis factor (TNF)-α levels have shown potential for indicating gingivitis and periodontitis.^9^ In addition, salivary levels of Toll-like receptor-4, IL-18, uric acid, aspartate transaminase and procalcitonin in patients with periodontitis were higher than in healthy individuals, showing positive correlations with clinical measurements including probing depth, clinical attachment level and gingival index. Hence, these parameters might be useful in the diagnosis and prognosis of periodontal diseases.^10, 11, 12^ Recently, a new system that uses a new type of oral rinse to estimate the neutrophil abundance in saliva was developed to screen for the presence of periodontal diseases. This system provides valuable information from the bench to chairside.^13^

### Oral cancer

The onset and development of malignancy are related to somatic mutations of tumour-specific DNA, which can be found in the saliva, plasma or other body fluids. These somatic mutations can be used as biomarkers to diagnose oral or other tumours. In saliva, tumour-specific DNA was positive in 100% of patients with oral tumours. However, only 47%–70% of patients with tumours in the other parts of the human body carry tumour-specific DNA in the saliva. In contrast, tumour-specific DNA was found in 80% of plasma samples from patients with oral tumours and in 86%–100% of patients with tumours in other sites. Based on these results, saliva has an overabundance of tumour-specific DNA from oral tumours. Thus, tumour-specific DNA in saliva has the potential to be applied to diagnose oral cancers.^14^ The DNA of tumour-related viruses in saliva, such as HIV and human herpes virus (HHV), can also be associated with cancers of the oral cavity or other sites.^15^ Park *et al.*^16^ found that miR-125a and miR-200a levels were of great significance as follows: compared with healthy individuals, patients with oral squamous cell carcinoma (OSCC) have lower levels in their saliva, indicating that miRNAs in saliva have a potential application in oral cancer detection.

Salivary proteins can also be used for cancer detection. It was reported that the increase in tumour antigen CA15-3 and antibodies for tumour protein markers c-erbB2, CA-125 and P53 in saliva can also be considered salivary biomarkers for cancers of the oral cavity and other sites.^17^ Further studies showed that hyaluronidase, IL-6 and IL-8 might be potential biomarkers for patients presenting with head and neck squamous cell carcinoma (HNSCC). Based on this study, they found that there was an overexpression of palate, lung, and nasal epithelium clone protein (PLUNC) and zinc-α-2-glycoprotein in the saliva of cancer patients. Both proteins might provide potential targets for a new analysis of HNSCC.^18^ Similar research showed that IL-4, IL-10, IL-13 and IL-1RA levels were increased in the saliva of patients with OSCC. Of note, the levels of IL-10 and IL-13 were significantly increased, whereas the level of IL-1RA was the highest in poorly differentiated OSCC lesions compared with well- and moderately differentiated OSCC lesions.^19^ A similar study found that the levels of TNF-α were higher in moderately and poorly differentiated tumours than in well-differentiated and stage IV tumours. Moreover, there was also a positive correlation between the histological grading of OSCC and TNF-α.^20^ In conclusion, salivary cytokines have been proven to be superior for detecting OSCC clinically and can be further investigated for use as biomarkers of histological grading and clinical staging for OSCC.

With the development of high-throughput sequencing technology, researchers have realized the importance of microorganisms in the development of oral cancer. The species diversity and relative abundance of bacteria in the saliva of patients with oral tumours are greater than in healthy patients.^21^

### Sjögren's syndrome

Sjögren's syndrome (SS) is a chronic systemic autoimmune disease characterized by keratoconjunctivitis sicca and xerostomia. With further development of SS, the salivary flow rate is decreased and the salivary constituents change. There are also significant changes in the proteome and transcriptome in patients with SS. Thus, the levels of IL-4, IL-5 and cytokine clusters might help to accurately predict the diagnoses for patients with SS.^22^ Another research identified 19 genes (*EPSTI1*, *IFI44*, *IFI44L*, *IFIT1*, *IFIT2*, *IFIT3*, *MX1*, *OAS1*, *SAMD9L*, *PSMB9*, *STAT1*, *HERC5*, *EV12B*, *CD53*, *SELL*, *HLA-DQA1*, *PTPRC*, *B2M* and *TAP2*) that were closely related to the pathological process of SS, which was characterized by functions such as induction of interferons, osmosis of lymphocytes and antigen presentation.^23^ Hu *et al.* successfully verified a panel of biomarkers that are elevated in patients with primary SS, including three mRNA biomarkers (myeloid cell nuclear differentiation antigen, guanylate binding protein 2 and low-affinity IIIb receptor for the Fc fragment of IgG) and three protein biomarkers (cathepsin D, α-enolase and β_2_-microglobulin).^24^ These biomarkers from the proteome and transcriptome might provide a simple clinical tool for the diagnosis of primary SS in the early stages.

## Diagnosis of systematic diseases by saliva

### Diabetes mellitus

Diabetes is a metabolic disease caused by insufficient insulin secretion, insulin action or insulin resistance, which leads to a glucose metabolism disorder. A positive correlation was found between α-2-macroglobulin and HbA1c, which demonstrated that levels of α-2-macroglobulin in the saliva could reflect the glycaemic control in patients with type 2 diabetes mellitus.^25^ However, the concentration of salivary melatonin decreased in patients with type 2 diabetes and patients with periodontitis. This indicates that salivary melatonin has an important role in the pathogenesis of diabetes and periodontal diseases, and might become a key biomarker in the diagnosis and treatment of these two diseases.^26^ Barnes *et al.*^27^ found 475 specific metabolites in the saliva of patients with periodontitis and/or diabetes. Levels of cellular energetic stress, purine degradation, glutathione metabolism, oxidized glutathione, cysteine glutathione disulphide, markers of oxidative stress, amino acids, a ω-3 fatty acid (docosapentaenoate) and ω-6 fatty acids (linoleate and arachidonate) signatures were significantly increased in patients who had gingivitis and periodontitis but not diabetes. In contrast, patients with diabetes had significantly higher levels of glucose and α-hydroxybutyrate, in addition to a significant change in the levels of carbohydrate, lipid and oxidative stress. Therefore, the metabolites might be useful for the diagnosis, treatment and prognostic assessment of periodontal diseases and diabetes. There was a significant correlation between both HbA1c and salivary glucose concentrations and patients with diabetes. Thus, this indicated that the blood glucose concentration could be monitored by the saliva in patients with diabetes mellitus.^28^

### Cardiovascular disease

Cardiovascular disease (CVD) is related to the circulatory system and includes atherosclerosis, myocardial infarction and coronary heart disease. Kosaka *et al.*^29^ found that levels of salivary inflammatory cytokines including IL-1β, IL-6, TNF-α and prostaglandin E2 increased significantly in both atherosclerosis and periodontal diseases. These cytokines might be potential biomarkers for the diagnosis of periodontal disease and atherosclerosis.^29^ Miller *et al.*^30^ identified that the C-reactive protein (CRP) was the most predictive biomarker of acute myocardial infarction. Acute myocardial infarction was predicted by a combination of electrocardiogram and CRP levels with 80.0% sensitivity and 100% specificity. These data demonstrated the potential use of salivary biomarkers with electrocardiogram for the diagnosis of acute myocardial infarction. Moreover, the levels of α-2-HS-glycoprotein in saliva decreased in patients with CVD, which indicates that the peptidome might provide a potential way for the early diagnosis of patients with CVD.^31^

### Viral infections

Diagnostic tests for viral infections currently rely on salivary biomarkers, such as viral DNA and RNA, antigens and antibodies. At the proteomic level, there are saliva-based antibody tests to detect viruses, including hepatitis A virus, hepatitis B virus, hepatitis C virus, HIV-1, measles virus, rubella virus and vesicular stomatitis virus mumps virus, among others. The Raffaele Scientific Institute in Milan used a new salivary test named OraQuick hepatitis C virus rapid antibody test, to detect the hepatitis C virus in an easier and faster way.^32^ Moreover, the dengue virus (DENV) RNA and non-structural protein 1 antigens are detectable from saliva, which might provide a more effective way to diagnose dengue.^33^ In the research of Nefzi *et al.*^34^ the saliva appears to be more sensitive than the blood in the detection of HHV-6 or human cytomegalovirus.

### Pancreatic cancer

Pancreatic cancer has a low incidence rate but a high mortality rate. Worldwide, more than 200 000 patients with pancreatic cancer are registered annually and the disease results in the death of 98% of patients. It has been predicted that pancreatic cancer will become the second cause of death worldwide by 2030.^35^ Therefore, it is important to diagnose and classify patients with pancreatic cancer at earlier stages, to give them timely treatment. In rodent models of pancreatic cancer, vesicles similar to exosomes can carry and transport tumour-specific biomarkers into the saliva.^36^ It was found that KRAS, MBD3L2, ACRV1 and DPM1 levels enabled the differentiation of patients with pancreatitis and healthy individuals.^37^ It was also found that hsa-miR-210 and let-7c were overexpressed in the saliva of patients with pancreatitis. In addition, significantly increased levels of hsa-miR-21, hsa-miR-23a, hsa-miR-23b, miR-29c and hsa-miR-216 were identified in the saliva of patients with pancreatic cancer; among them, hsa-miR-23a and hsa-miR23b were overexpressed in precursor lesions.^38^ Moreover, miR-3679-5p and miR-940 have an excellent ability to indicate pancreatic cancer, which enables curative surgery.^39^ The results of an ROC-plot AUC can distinguish patients with pancreatic cancer from chronic pancreatitis and healthy individuals with a 90.0% sensitivity and 95.0% specificity.^40^

There are also correlations between periodontitis and pancreatic cancer onset. Patients with periodontitis had a 64% higher risk of pancreatic cancer.^41^ There was an increase in 31 bacterial species and a decrease in 25 bacterial species in the saliva of pancreatic cancer patients. Moreover, two bacterial biomarkers, *Neisseria elongata* and *Streptococcus mitis*, have a high sensitivity and specificity for the diagnosis of patients with pancreatic cancer.^42^

### Breast cancer

Breast cancer is one of the most common cancers in females. ATP6AP1 is an ATPase that is expressed in normal tissues such as the brain marrow, blood, nerves and skin, and it is also correlated with several tumours such as head and neck carcinomas, lung tumours, adrenal tumours and various other cancers. Nevertheless, its prevalence in breast cancer is the greatest among these cancers. ATP6AP1 auto antibodies are spontaneously generated in patients and they can be detected in early stages. Thus, it is indicated that ATP6AP1 can contribute to the early detection of breast cancer.^43^ Zhang *et al.*^44^ found eight mRNA biomarkers and one protein biomarker that could be used to detect breast cancer with an 83% sensitivity and 97% specificity. In another study, it was found that the levels of vascular endothelial growth factor, epidermal growth factor (EGF) and carcinoembryonic antigen in the saliva were significantly increased in patients with breast cancer.^45^ The levels of CA15-3 and c-erB-2 were also found to be increased in the saliva, which has positive correlations with the serum of patients with breast cancer.^46^ Based on these studies, potential salivary biomarkers can be applied to the early diagnosis of breast cancer.

### Lung cancer

Lung cancer has a high incidence rate. Mutations identified in the EGF receptor (EGFR) are the tumour-specific biomarkers for non-small cell lung carcinoma (NSCLC). A novel core technology known as electric field-induced release and measurement relies on a multiplexible electrochemical sensor that can detect EGFR mutations in bodily fluids was shown to be effective, accurate, rapid and cost-effective for the detection of EGFR mutations in the saliva of patients with NSCLC.^47^ In addition, Xiao *et al.*^48^ found 16 candidate proteins that can discriminate lung cancer patients from healthy individuals and are effective biomarkers with a high sensitivity and specificity. This demonstrates that proteomic biomarkers can be established for the early detection and prognosis of lung cancer.

### Prostate cancer

MiR-141 and miR-21 are two tumour biomarkers; the former is significantly elevated in patients with advanced-stage prostate cancer, whereas the latter is overexpressed in early-stage prostate cancer. It has been demonstrated that the expression of miR-21 and miR-141 in the saliva can be detected by nano-graphene oxide. This is expected to be a non- or minimally invasive approach to diagnose early-stage prostate cancer.^49^

### Other diseases

In the saliva of patients with gastric ulcer and chronic gastritis, *Helicobacter pylori* DNA can be detected to identify *H. pylori* infection.^50^ Significant correlations were found between salivary caffeine clearance and liver diseases. Thus, saliva can be used as an effective biochemical parameter for the diagnosis of chronic liver diseases (CLDs) and assessment of residual liver function in CLD.^51^ The saliva of patients with chronic renal failure presented significantly higher levels of NO. After haemodialysis treatment, the saliva showed significantly higher levels of immunoglobulin (Ig)A, IgG and CRP. Thus, this demonstrated that salivary levels of IgA, IgG, NO and CRP might have an important role in monitoring renal disease.^52^ Further studies found significant associations between fusion transcript levels in the saliva and bone marrow. Such associations might allow for the potential use of saliva to detect minimal residual disease in leukaemia.^53^ Based on recent studies, it has been proposed that salivary trehalose, which could be detected by some cell-based extended gate ion-sensitive field-effect transistor biosensors, might provide a sensitive and direct way to screen for Alzheimer's disease.^54^ Wilson's disease is a rare inherited disorder of copper metabolism and is characterized by hepatic, neurological and psychiatric symptoms. Researchers analysed the salivary proteome and peptidome of Wilson's disease patients and found an increase in α-defensins 2 and 4. Overall, the oxidative stress and inflammatory conditions reflected by the salivary proteome of patients with Wilson's disease might be key clues of disease exacerbation.^55^ Moreover, the level of salivary cortisol is a regular biomarker for psychology stress. Three potential biomarkers from saliva have been demonstrated to diagnose atopic dermatitis in its early stage.^56^

## Problems

The advantages of saliva as a diagnostic fluid are that it is simple to collect, convenient to store, essentially non-invasive and contains high-quality DNA. Thus, we conclude that saliva is a perfect substitute for blood. The research in salivaomics has an important role in identifying biomarkers of diseases and potential drug targets. Salivaomics also has the potential to diagnose diseases in their early stages. However, research on saliva and its applications for the diagnosis of disease is still in its early stages and the progress of these studies is limited by the lack of efficient and useful methods and techniques. We need to develop systems of salivary molecular identification and standardize salivary evaluation. Systemic knowledge networks of salivaomics and precise biomarkers of diseases can contribute to a better understanding of the correlations between oral health and systemic health, which will promote the application of precision medicine by facilitating the individualization of precise, pain-free and convenient targeted therapy.
